# The use of Savary-Gilliard dilators in the treatment of an oesophageal stricture in a cat

**DOI:** 10.1007/s11259-022-09891-x

**Published:** 2022-02-02

**Authors:** Ewa Kaczmar, Krystyna Makowska, Andrzej Rychlik

**Affiliations:** Department of Clinical Diagnostics, Faculty of Veterinary Medicine, Uniwersity of Warmia and Masury in Olsztyn, Oczapowskiego 14, 10-957 Olsztyn, Poland

**Keywords:** Endoscopy, Cat, Savary-Gilliard dilators, Bougienage, Oesophageal stricture, Oesophagus

## Abstract

Oesophageal strictures in cats and dogs are relatively rare and the cause of this disorder can be multifactorial. However, the most common cause in cats is an inflammatory process.

Conservative treatment strategies for this disorder includes image-guided interventions. Endoscopic methods are a form of a minimally invasive surgical treatment of the oesophageal strictures. Several endoscopic methods for the therapy of this condition are known, one of them is Savary-Gilliard dilators technique.

In the present study of a case of oesophageal stricture in a cat, caused probably by doxycycline treatment without water administration, the authors used the Savary-Gilliard dilators as a therapy for its condition. The animal underwent 3 endoscopy procedures, where in the third one no abnormality in the oesophagus was observed. Moreover, the cat was asymptomatic 6 months after the last oesophagoscopy.

In the authors opinion, based on the present case, some experience of the authors and previously described studies, the Savary-Gilliard dilators seems to be a safe, effective, relatively cheap and minimally invasive method of the oesophageal stricture therapy in the cat.

## Introduction

Oesophageal strictures in cats and dogs are relatively rare disorders consisting in a significant narrowing of the esophageal lumen (Adamama-Moraitou et al. 2002; Glazer and Walters 2008) associated with damage to the mucosa, reaching the submucosal and/or muscular layers of the oesophageal wall. Chronic inflammation or ulcers extending through the lamina propria to the muscle layer result in the formation of fibrous and scar tissue, which in the healing process cause the stricture (Zawie 1989; Adamama-Moraitou et al. 2002).

Clinical signs of an oesophageal stricture in animals are nonspecific. The main features are due to dysphagia which firstly affect the ingestion of large portions of solid food, but later they can include semi-solid and liquid food as well (Adamama-Moraitou et al. 2002; Gallagher and Specht 2013). The characteristic sign of an oesophageal stricture—regurgitation, is secondary to the accumulation of food in the oesophagus. It is usually an effect of the oesophageal dilatation occurring proximally to the stricture (Sellon and Willard 2003).

Oesophageal strictures are usually diagnosed on the basis of a signalment, history, the results of clinical examination, and radiography, but the definitive diagnosis is often made by the oesophagoscopy (Glazer and Walters 2008). It is now believed that although contrast-enhanced radiography is a good method for the diagnosis of oesophageal strictures, the endoscopic examination gives much more information about the diameter of the lumen and the pathological changes in the oesophageal mucosa. Moreover, oesophagoscopy offers therapeutic possibilities, which makes this examination the method of choice to confirm oesophageal strictures (Sullivan and Miller 1985; Adamama-Moraitou et al. 2002). On the other hand it is important to note that the use of both radiography and endoscopy helps to precisely determine, whether the stricture is due to the oesophageal disease, or whether the changes in the oesophageal wall are secondary to other, extramural diseases.

Treatment strategies of the oesophageal stricture in dogs and cats are mainly image-guided interventions, including ballooning, the Savary-Gilliard dilators technique (bougienage) and stenting (Galatos et al. 1994; Adamama-Moraitou et al. 2002; Couturier and Guilbaud 2006; Bissett et al. 2009; Gallagher and Specht 2013; Tan et al. 2018).

The present paper describes the case of application of minimally invasive procedure with the Savary-Gilliard dilators as an effective treatment method of the oesophageal stricture in a cat.

## Material and Methods

### Case Presentation

A 3-year-old female cat was found with the signs of ataxia and abnormal gait. Clinical examination reveals that the animal was severely dehydrated and hypothermic (body temperature 36,5^0^C) with a tense and painful abdomen. The palpation of the abdomen suggests the thickening of intestines. Additional laboratory tests including complete blood cell count, serum biochemical analysis, urinalysis, feline immunodeficiency virus and feline leukemia virus tests and radiographic and ultrasound imaging of the abdomen were performed. The results revealed leukopenia. The FIV/FeLV test was negative. Radiographic images showed gas in the stomach and intestines, and ultrasonography showed thickening of intestines with no intra-abdominal free fluid. Finally a parvovirus antigen test (Idexx Parvo SNAP test) was taken and the result was positive, so the cat was diagnosed with feline panleukopenia.

The patient was treated with intravenous fluid therapy (3 to 4,4% of cat weight per day) to correct dehydratation and intensive supportive care. Antibiotics (full spectrum: enrofloxacin (Baytril 5 mg/kg SID) intravenously and an injectable penicillin drug (Penicillin 20,000 units per kg BID or SID) and to prevent fever—nonsteroidal anti-inflammatory drugs (Metacam 0,3 mg/kg SID). Because the patients condition did not improve, doxycycline (5 mg/kg BID orally) was added to the treatment.

After these treatments, the patient's clinical condition improved. However, after 2 weeks some new gastrointestinal signs, including the anorexia, slight diarrhea, salivation, choking, dysphagia and regurgitation and occacionally vomit were observed. Then, the patient received intravenous fluid therapy (3 to 4,4% of cat weight per day) and some drugs such as: maropitant (Cerenia in a dose of 1 mg/kg SID), omeoprasol (0,5 mg/kg SID), vitamin and mineral supplement (Vetaminex SID) and probiotics (Bioprotect SID) have been added for the treatment of the patient however, it’s condition did not fully improved. After couple days of the treatment the patient was stronger, without diarrhea and vomits however, anorexia, salivation, dysphagia, choking and regurgitation were still observed. After a radiographic examination with barium contrast which showed gas in the stomach and intestines and a residue of contrast in the esophagus the animal underwent endoscopic examination.

### Endoscopy

Food was withheld for 12 h prior to the procedure and the water was withheld an 1 h before the start of general anesthesia. The animal was premedicated intramuscularly with medetomidine hydrochloride (Domitor 1 mg/ml, Orion Corporation, Finland; 0,02 mg/kg body weight (b.w.)) and butorphanol tartrate (Butomidor 10 mg/ml, Richter Pharma AG, Austria; 0,025 mg/kg b.w.) and then induced with propofol intravenously (Provive 10 mg/ml, Claris Lifesciences (UK) Ltd., United Kingdom; 6 mg/kg b.w).

**Fig. 1:**
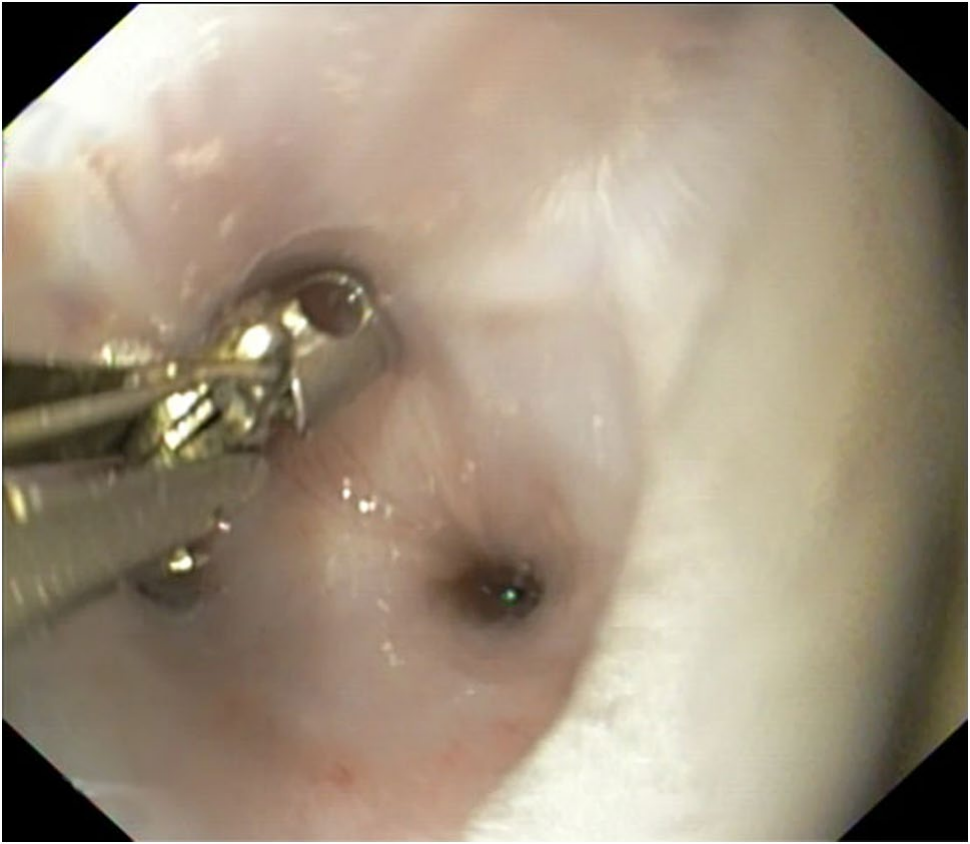
The stricture (with a diameter less than 2 mm) in the proximal segment of the oesophagus found during the first oesophagoscopy. The biopsy forceps have a cup diameter of 7 mm

**Fig. 2:**
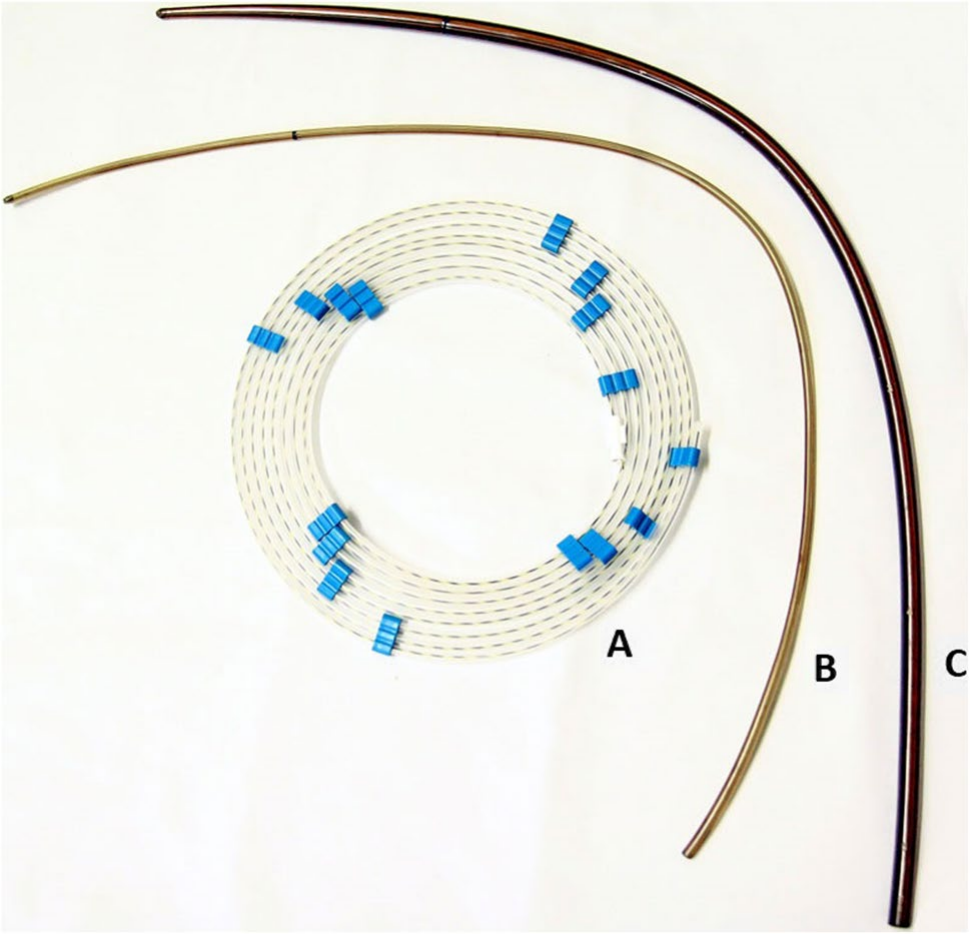
A Savary-Gilliard dilation set

During the examination the patient was intubated and then positioned in left lateral recumbency. Oesophagoscopy was performed using a flexible videoendoscope (Olympus type GIF-Q145, Olympus Inc., Hamburg, Germany) with a working length of 1030 mm and a diameter of 9.8 mm. Endoscopy revealed the stricture of the lumen in the cervical segment of the oesophagus with the diameter of the oesophageal lumen reduced to 2 mm. (Fig. 1). After the diagnosis, a Savary-Gilliard expansion set consisting of a special wire guide with a soft rubber tip to prevent injuries and perforation of the mucosal layer and a plastic dilator firstly of 5 mm, then another with 10 mm diameter (at its widest part) was used to dilate the oesophagus (Fig. 2). This treatment was performed according to the method previously described by Bisset et al. 2009 (Bissett et al. 2009). In brief, the procedure was performed as follows: first, the endoscopic guide was inserted through the working channel of the endoscope. After passing the guide, via the endoscope working channel, through the stricture into the larger space of the stomach the endoscope was withdrawn, leaving the guide traversing the stricture. At this point, there was no longer a visual inspection, so the dilator was passed over the endoscopic guide wire (the dilator has an internal channel along its length). Before the dilator was inserted into the oesophagus it was covered with a lubricant. When inserting the dilator through the stricture, a slight resistance was encountered, which had to be overcome by gently moving the dilator backward and forward.. After the procedure the animal was protected against the risk of postoperative complications by continuing routine antibiotic and anti-inflammatory therapy.

Since mentioned above, the procedure was minimally invasive, the animal was in good clinical condition, but the gastrointestinal signs relapsed after 2 weeks..

**Fig. 3:**
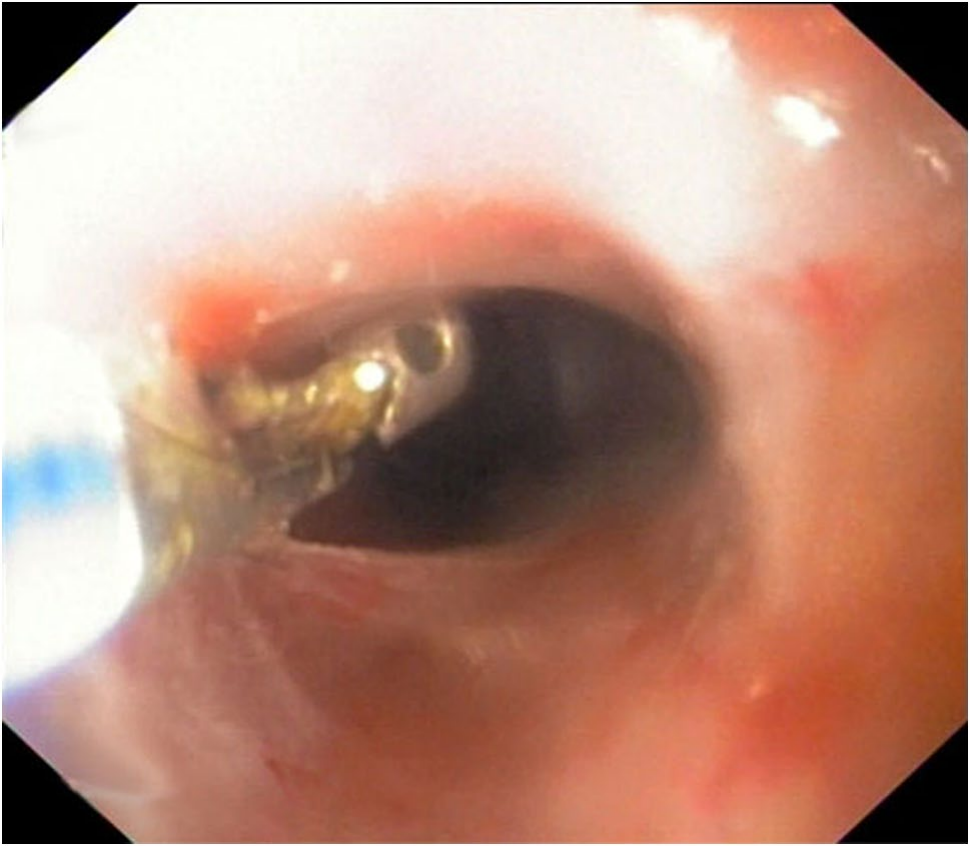
Endoscopic view showing the oesophageal diameter after the second procedure. The forceps have a cup diameter of 7 mm

A follow-up oesophagoscopy was carried out after 2 weeks. The examination showed recurrence of 3 mm diameter stricture (Fig. 3). The procedure of oesophagus dilation with the use of Savary-Gilliard dilator was repeated in the same way as previously.

**Fig. 4:**
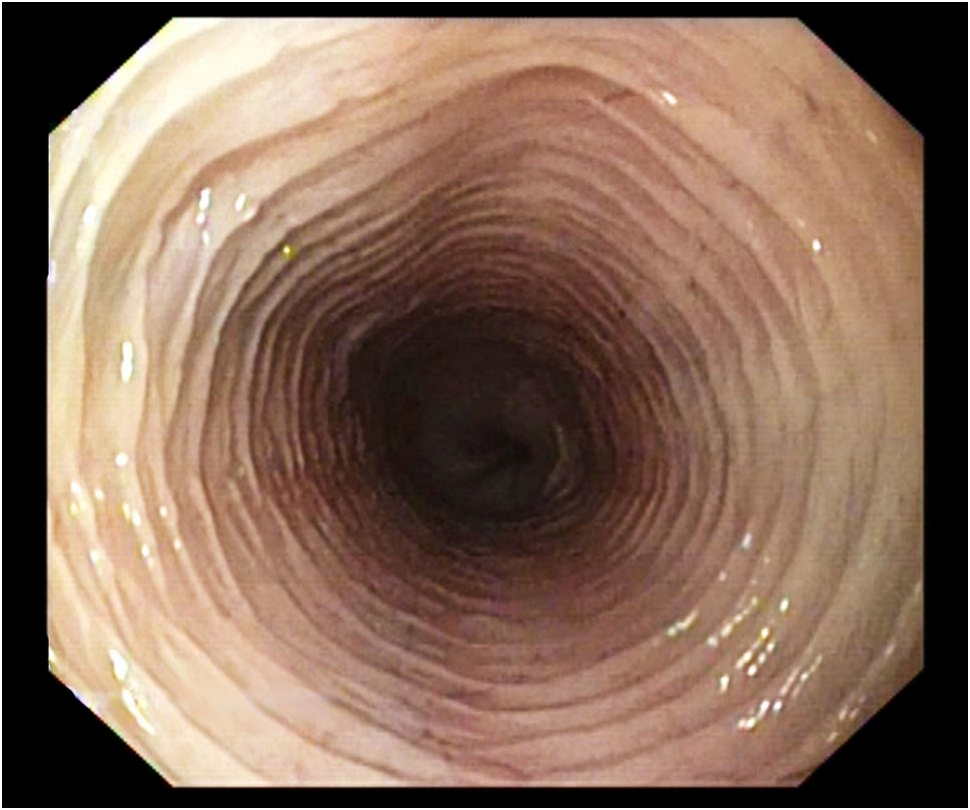
The image taken during the third oesophagoscopy. No abnormalities in the oesophagus anatomy were found

The next endoscopic examination of the patient was performed after another 4 weeks. Till this time the animal gained almost 1 kg weight. During this endoscopy, no oesophageal abnormalities were found (Fig. 4). Since then the animal has no longer regurgitated, but the cat owner continue to use only semi-solid diet. According to the follow-up information the cat was asymptomatic up to 6 months after the last oesophagoscopy.

## Discussion

The oesophageal stricture is a rare condition, and due to the nonspecific signs, rarity of occurrence and not very common use of endoscopy in veterinary clinical diagnostics very frustrating and difficult to manage for clinicians and owners (Willard 2004).

Oesophageal strictures are often secondary to an inflammatory process. Moreover, previous studies have also reported that the oesophageal strictures in both, humans and animals can occur after oral administration of tetracyclines (specifically doxycycline tablets), as it most probably was in the presented case (Carlborg and Densert 1980; Carlborg and Densert 1983; Bissett et al. 2009; McGrotty and Knottenbelt 2002; Willard 2004).

The results of previously described experimental study have shown that doxycycline and oxytetracycline administrations produced areas of deep ulceration in the oesophagus (Carlborg and Densert 1980; McGrotty and Knottenbelt 2002). Moreover, doxycycline capsules, not tablets were mostly associated with oesophageal damage. This observation was probably associated with a longer retention time in the oesophagus of this form of drugs (Carlborg and Densert 1983; McGrotty and Knottenbelt 2002). Furthermore, the oesophageal retention time can be minimized by administering capsules and tablets with food or providing large amount of water after the drug administration (McGrotty and Knottenbelt 2002).

During the described case the patient was treated with several drugs due to the diagnosed panleukopenia. The patient was treated with enrofloxacin intravenously and an injectable penicillin drug. This treatment strategy did not improve the patient's condition so it was changed for tetracyclines (mostly because of a broad spectrum of activity and action against gram – bacteria). Most probably the cause of the oesophageal stricture in the present case was long-term doxycycline treatment without water administration as described previously (German et al. 2005; Sasaki et al. 2017). Unfortunately, due to better access to doxycycline tablets and the lack of possibility of daily intravenous administration of drugs for a long time, it was decided to use this solution, despite the risk of oesophageal stricture. The owner was informed about the need to give large amounts of water or food after taking the tablet, but it is most likely that this practice was not fully followed.

In veterinary medicine several treatment methods can be used in the cases of oesophageal strictures. The conservative treatment relies on special, semi-liquid diet, high positioning of the bowl and treating the underlying disease (Wesdorp and Bartelsman 1982; Gallagher and Specht 2013). Moreover, the oesophageal stricture can be treated pharmacologically with the anti-inflammatory drugs (including steroids) and antibiotics. This method is extrapolated from human medicine, but its effectiveness in veterinary patients has not been confirmed yet (Yan et al. 2019). Finally, the oesophageal stricture may be resolved mechanically that is through surgical operation or endoscopy. Surgery includes resection and anastomosis, oesophagoplasty or reconstructive procedures (patch grafting), and is indicated when conservative treatment fails or in the case of neoplastic or tubular strictures. Surgery can be an effective method of the treatment for the oesophageal stricture, but it is not commonly performed due to the complexity of the treatment, the specialized surgical equipment requirements, and the potentially poor outcome (Johnson et al. 1992; Fingeroth 1993).

Therefore the endoscopic methods, which are less risky and less invasive than surgical operation and more effective than drug therapy are the preferred methods of treating oesophageal strictures in dogs and cats. However, the procedures involving the mechanical expansion of oesophageal strictures may need to be performed several times to consolidate the effect, which is associated with the need of repeated anesthesia and great discomfort for the patient (Adamama-Moraitou et al. 2002; Bissett et al. 2009; Gallagher and Specht 2013). Therefore, the use of stents seems to be a preferable alternative. Unfortunately, the high costs associated with the purchase of stents and the lack of a guarantee that it will not move or be expelled, so far does not favour the use of this method for treatment of pets (Tan et al. 2018).

The most common endoscopic method for managing the oesophageal stricture is using dilatating balloons. It is important to note that balloon dilation is considered more effective in some cases due to the better control of the dilating segment of the oesophagus (Chae et al., 2021). On the other hand, cons of this method are mostly its high costs in some countries, the difficulties in properly matching the right size of the balloon and the need to use appropriate pressure during the procedure. Moreover, ideally, the use of the dilating balloons should be performed with additional imaging techniques like fluoroscopy. There are only few reports regarding the use of Savary-Gilliard dilators in similar cases in cat patients (Bissett et al. 2009). Nevertheless, the bougienage technique with Savary-Gilliard dilators seemed to be an effective method of treatment of oesophageal strictures in cats (Bissett et al. 2009), which is also confirmed by the case described above. Pros of the bougienage technique are first of all its low costs (the equipment is reusable) and high availability in some countries. It should however be pointed out, that for the proper use of this method a presence of well experienced endoscopist is essential.

Although the stricture was improved after the procedure there is a possibility that the patient spontaneously recovered from the condition. A spontaneous recovery in oesophageal stricture in cats, according to the authors experience is very rare. Due to the patient's medical history it is unlikely that could happened in this case, nevertheless, because of the lack of image data other than the photograph obtained during the endoscopy it is a limitation to the presented study.

## Conclusion

Tetracyclines (especially doxycycline tablets) should, whenever possible, be administered with provided water or food after the administration of the drug to minimize the risk of oesophageal damage. Both, veterinary surgeons and cat owners should be aware of the potential risk for oesophageal damage associated with oral treatment with tetracyclines.

Moreover, the use of the Savary-Gilliard dilators seems to be a safe, effective, relatively cheap and minimally invasive method of the oesophageal stricture therapy in the cat. Therefore it could be used more often as an alternative for the technique with the traditional dilating balloons.

## Conflicts of interest

The authors declare no conflict of interests.

## Statement of Animal Ethics

not applicable.

## Ethics approval

not applicable.

## Consent to participate

not applicable.

## Consent for publication

not applicable.

## Data Availability

All data generated or analysed during this study are included in this published article.
